# Exosomal miR-767 from senescent endothelial-derived accelerating skin fibroblasts aging via inhibiting TAB1

**DOI:** 10.1007/s10735-022-10107-4

**Published:** 2022-11-21

**Authors:** Jing Li, Jiyong Tan, Qiong Song, Xinni Yang, Xin Zhang, Hao Qin, Gaoxiang Huang, Xiaoxue Su, Jing Li

**Affiliations:** 1 Department of Physiology, Guangxi Medical University, Nanning, 530000 Guangxi China; 2 Key Laboratory of Longevity and Aging-Related Diseases, Ministry of Education, Nanning, 530000 Guangxi China; 3 Guigang City People′s Hospital, Guigang, 537000 Guangxi China

**Keywords:** Exosome, miR-767, TAB1, Aging, Fibroblasts

## Abstract

Skin aging is a complicated physiological process, and microRNA-mediated regulation has been shown to contribute to this process. Exosomes mediate intercellular communication through miRNAs, mRNAs and proteins, and participate in many physiological and pathological processes. Vascular endothelial cell-derived exosomes have been confirmed to be involved in the development of many diseases, however, their effects on skin aging have not been reported. In this study, senescent endothelial cells could regulate skin fibroblast functions and promote cell senescence through exosomal pathway. miR-767 was highly expressed in senescent vascular endothelial cells and their exosomes, and miR-767 is also upregulated in skin fibroblasts after treatment with exosomes derived from senescent vascular endothelial cells. In addition, transfection with miR-767 mimic promoted senescence of skin fibroblasts, while transfection with miR-767 inhibitor reversed the effect of D-galactose. Double luciferase analysis confirmed that TAB1 was a direct target gene of miR-767. Furthermore, miR-767 expression was increased and TAB1 expression was decreased in D-galactose induced aging mice. In mice that overexpressed miR-767, HE staining showed thinning of dermis and senescence appearance. In conclusion, senescent vascular endothelial cell-derived exosome mediated miR-767 regulates skin fibroblasts through the exosome pathway. Our study reveals the role of vascular endothelial cell-derived exosomes in aging in the skin microenvironment and contributes to the discovery of new targets for delaying senescence.

## Introduction

Aging is a biological law in nature, the common hallmarks of aging are genomic instability, telomere attrition, epigenetic alterations, loss of proteostasis, deregulated nutrient-sensing, mitochondrial dysfunction, cellular senescence, stem cell exhaustion, and altered intercellular communication(López-Otín et al. 2013). Skin aging is an intuitive manifestation of aging, and its causes are mainly divided into internal and external causes. The intrinsic factors often refer to the aging of the body caused by genetic and physiological function decline. It is characterized by decreased proliferation of basal cells and accumulation of senescent cells in the epidermis and dermis (Ansary et al. 2021). The external factors usually refer to skin aging caused by environmental factors, such as ultraviolet rays, chemical irritation, active oxygen damage, etc. (Lephart. 2016). The dermis is mainly composed of fibroblasts, which play a role in most of the features of skin aging, either directly or through interactions with other cells (Gruber et al. 2020). Relevant studies show that endothelial cells can participate in the regulation of skin fibroblast function. For example, in the co-culture system of skin microvascular endothelial cells and dermal fibroblasts, the decrease in cell density caused by hypoxia is more moderate compared with that of cultured fibroblasts alone (Oberringer et al. 2008). And in the animal model of endothelin-1 knockout, it was found that endothelin-1 knockout could alleviate bleomycin induced skin fibrosis (Makino et al. 2014). Endothelial cells-derived-extracellular vesicles treatment enhanced fibroblast proliferation, and decreased senescence through the elevation of YAP nuclear translocation and activation the PI3K/Akt/mTOR pathway (Wei et al. 2020). The specific molecular mechanism of the information transmission regulation between endothelial cells and skin fibroblasts has not been clarified, especially the relationship between aging endothelial cells and fibroblasts, which needs further study.

Exosomes are small membrane particles with a size of 40–150nm, originated from endoplasmic bodies, and play a key role in intercellular communication by transferring miRNAs, mRNAs and proteins to recipient cells (Miao et al. 2021). Cells of different germ lines can exhibit changes in exosome secretion under normal and abnormal pathological conditions, and exert certain biological activities. For example, extracellular vesicles secreted by stem cells from different sources can control inflammation, accelerate the migration and proliferation of skin cells, improve angiogenesis, and even improve signs of skin aging (Tkach et al. 2016). Exosomes from human umbilical cord blood can mediate miR-21-3p to promote the proliferation and migration of fibroblasts, and enhance the angiogenic activity of endothelial cells to promote skin wound healing (Hu et al. 2018). In addition, studies have shown that aging dermal fibroblasts release more exosomes than resting human skin fibroblasts, and all miRNAs per cell increase by 80% (Terlecki-Zaniewicz et al. 2018).

miRNAs are a type of non-coding single-stranded small-molecule RNA with a length of about 22 nucleotides encoded by endogenous genes, which affects the gene expression process by combining 3 'non-coding regions. The role of miRNA in gene regulation pathways is a key step in many biological processes, including development and cell signal transduction, proliferation, differentiation, apoptosis and aging. Studies have shown that the expression profile of miRNA has changed between health and disease, so miRNA may be considered a biomarker of disease (Alipoor et al. 2016). There is evidence that miRNA is also involved in the regulation of aging-related processes, such as: miR-200b-3p and miR-200c-3p regulate the cell cycle by targeting HMGB3 (Liu et al. 2018). miR-101 regulates Ezh2 to induce senescence in human fibroblasts (Greussing et al. 2013). miR-27a-5p-SMAD2-MMMP1/COL1/BCL2 axis can increase apoptosis and induce cell arrest in G2/M phase in human skin primary fibroblasts (Jiang et al. 2020).

Some studies believe that exosomes derived from vascular endothelial cells can regulate ischemic kidney injury by releasing miR-486-5p (Viñas et al. 2016). The exosome miR-34a derived from skeletal muscle cells in the circulating blood gradually increases during aging, and exosome miR-34a can also cause the senescence of bone marrow mesenchymal stem cells (Fulzele et al. 2019). More interestingly, exosomes derived from bone marrow mesenchymal stem cells regulate aging-related insulin resistance by mediating miR-29b-3p (Su et al. 2019). And exosomes derived from senescent osteoblasts promote senescence and apoptosis of vascular endothelial cells and inhibit their proliferation and migration by mediating miR-139-5p (Lu et al. 2021). We hypothesized that vascular endothelial cells mediate miRNA regulation of skin fibroblasts through exosomes during aging. From the perspective of exosomes, we explored the role of endothelial cell-derived miRNAs in regulating the aging of skin fibroblasts, and further explored the molecular mechanism of skin aging.

In this study, we explored the role of aging endothelial cell-derived miRNAs in regulating the aging of skin fibroblasts, and further explored the molecular mechanism of skin aging.

## Materials and methods

### Cell culture and co-cultivation system

Mouse skin firoblasts (MSFs) from 5–7 days Kunming suckling mice (provided by Animal Experimental Center of Guangxi Medical University) were digested by 0.1% type I collagenase enzymes for 1.5 h. Placed in Dulbecco’s modified Eagle’s medium (DMEM) (GIBCO, CA, USA) containing 10% fetal bovine serum (FBS) (GIBCO, CA, USA) at 37 °C in thermostatic incubator with 5% CO_2_ for culture. Vascular endothelial cells (Aolu, Shanghai, China) were seeded into 10 cm culture dish with a density of 1 × 10^6^ cells. After 24 h of incubation, add cell culture medium containing D-galactose (D-gal, 20 g/L) for 48 h. A co-cultivation system of endothelial cells and skin fibroblasts was established by using the Transwell chamber and culture for 48 h. Vascular endothelial cells were plated in upper chambers, and skin fibroblasts were grown on the lower chamber.

### Animal experiment

In total, 30 C57 mice were purchased from experimental Animal Center of Guangxi Medical University and randomly divided into control group (CTRL group) and experimental group (Aging group). CTRL group received subcutaneous injection of 0.9% sodium chloride injection in the neck and back. Aging group was injected with D-gal solution (250 mg/kg) once a day, for three months. The mice were weighed once a month, and the dose was adjusted according to their body weight. After three months, the skin was taken for further experiment.

Four C57 mice were purchased from Experimental Animal Center of Guangxi Medical University. Randomly divided into NC group (Back injection equal 0.9% sodium chloride injection) and miR-767 agomir group (back injection of 70 μl miR-767 mimics (5 μg/μl) + 130 μl PBS). The mmu-miR-767 mimics were synthesized by GenePharma Co., Ltd. (Suzhou, China). Once a week, skin was collected after four injections.

### Cell transfection

The MSFs were transfected with miR-767 mimics, inhibitor, and negative control (NC) (RiboBio, Guangzhou, China) to up-regulate or down-regulate miR-767 expression, respectively. Then, si-001, si-002, and si003 (si-TAB1) (RiboBio, Guangzhou, Shanghai) were transfected into the MSFs. Cells were harvested 48 h after transfection for further experiments.

### RNA extraction and quantitative real-time-PCR (qRT-PCR)

Total RNA was extracted from cells using Trizol reagent (Thermo Corporation, USA) according to the manufacturer’s instructions. Reverse transcription was performed using Biotechnology (Shanghai) company’s microRNA First-Strand cDNA Synthesis Kit and RevertAid First Strand cDNA Synthesis Kit. Analysis of mRNA expression by qRT-PCR using Power SYBR Green PCR Master Mix (Invitrogen, US). qRT‐PCR was completed on ABI Prism 7300 RT PCR System. The expression levels of miR-767 and U6 were determined using microRNA First-Strand cDNA Synthesis Kit, with U6 as the internal control. The upstream primer was designed and completed by Shanghai Sangon Biotech Company. Relative mRNA expression was shown as fold change (2 − ΔΔCt) with GAPDH as internal control. The primers are showed in Table 1.

**Table 1:** Primer sequences used for Qμantitative real-time-PCR

Primer name	Primer sequence
mmu-miR-767	UGCACCAUGGUUGUCUGAGCA
TAB1	F: GTCGTGGCAGTCCTTCTCAACAG
	R: TCGTCCTCGTTCTCGGTGGTG
GAPDH	F: GGTTGTCTCCTGCGACTTCA
	R: TGGTCCAGGGTTTCTTACTCC

### Exosome isolation and exosomal RNA extraction

Collect the cell culture supernatant and centrifuge at 3000 g for 15 min to remove cell debris. 1 ml ExoQuick-TC were mixed with 5 ml medium at 4 °C overnight. Exosomes were extracted from the supernatant using the SBI ExoQuick TC Ultra EV separation kit (System Biosciences, USA), according to the manufacturer’s instructions. After isolating exosomes from cells, 350 ul LYSIS buffer and 200 ul absolute ethanol were added to the exosomal pellet. The precipitation was obtained by using a centrifuge column and a collection tube, to which 30ul ELUTION buffer was added and placed into a 1.5 mL elution tube without RNase for centrifugation at 2000 rpm for 2 min, then increasing the rate to 13,000 rpm for 1 min to obtain RNA.

### β‐galactosidase staining

According to the manufacturer’s descriptions, the β-galactosidase assay was performed by β-galactosidase Cell Senescence Staining Kit (Beyotime Biotechnology Company). After washing and fixing, cells were incubated at 37 °C overnight with β-galactosidase stain solution and images were collected by microscope (Olympus, Japan). The percentage of β-gal staining positive cells out of the total number of cells was counted and the mean percentage was obtained from three independent experiments.

### Hoechst staining

Hoechst staining was performed with Hoechst Cell Apoptosis Staining Kit (Beyotime Biotechnology Company). After washing and fixing, cells were stained with Hoechst 33,258 for 5 min, then washed twice with PBS. Drop anti-fluorescence quenching mount solution, then apoptotic nuclei (blue fluorescence) can be observed by fluorescence inverted microscope (Olympus, Japan).

### Ki67 staining

Cells were cultured in 6-well plates for Ki67 staining with the Ki67 Cell Proliferation Staining Kit (Sangon Biotech Company, Shanghai). After immobilized with paraformaldehyde (4%), permeated with 0.1%-0.5% Triton X-100 membrane, and sealed with blocking solution, each sample was incubated with 50μL Cy3-conjugated Goat Anti-Rabbit IgG (Component A, diluted at 1:50–1:200) and incubated for 60 min in dark at room temperature. The staining was observed under a fluorescence inverted microscope (Olympus, Japan) at wavelength of 550 nm.

### Dual‐luciferase reporter assay

The TAB1 3’-UTR wild-type (WT) and mutant type (MT) sequences of the target gene, TAB1, were cloned into the pSICheck-2 plasmid (Sangon Biotech Company, Shanghai, China) to establish TAB1-WT and TAB1-MT plasmids. The plasmids were co-transfected with miRNA mimic or negative control mimic into cells. 48 h after transfection, cells were collected and luciferase activity was detected by Dual‐Luciferase Reporter Gene Assay Kit.

### Western blot analysis (WB)

Cells were washed with iced PBS and then harvested in RIPA lysis buffer containing protease inhibitors cocktail (Solarbio Bio). Determine protein concentration by BCA protein determination kit (Beyotime Biotechnology Company). A total of 45 μg protein extracts were loaded, then separated by 10% SDS polyacrylamide gel electrophoresis, and transferred on polyvinylidene difluoride (PVDF) membranes (Millipore, USA). Blocked in TBST in 5% milk at room temperature for 1 h, the membranes were incubated with the Anti-TAB1 monoclonal antibodies (ABCAM company, UK) overnight at 4 °C, and then incubated with either HRP-labeled sheep anti-rabbit secondary antibodies or HRP-labeled sheep anti-mouse secondary antibodies. The signal was detected by chemiluminescence reagent (TAKARA), and the protein band was detected by a chemiluminescence detection system, which was analyzed by Image-Pro Plus 6.0. GAPDH as internal control.

### Hematoxylin–eosin (HE) staining

Mouse skin samples with 4% paraformaldehyde fixed liquid fixed, paraffin embedding, sectioning (thickness 5 μm), dimethyl benzene dewaxing, gradient ethanol dehydration, hematoxylin staining 6 min, wash to dye, hydrochloric acid ethanol differentiation for a few seconds, rinse slices returned to blue, eosin staining for 1 min, the ethanol dehydration. With a neutral gum sealing piece, and observed under a microscope. Using ImageJ software, the skin thickness was calculated in μm digital form and measured ≥ 3 times from the epidermis to the dermal fat junction.

### Masson’s trichrome staining

According to the manufacturer's instructions, the skin of mice was stained with the Masson’s trichrome staining kit (Solarbio, Beijing, China). Mouse skin Sects. (5 μm) were routinely dewaxed and rehydrated, stained with Hematoxylin iron solution Weigert for 15 min, and rinsed with water until the nuclei turned dark blue. Staining in Ponceau solution for 10 min, rinsing with 1%HAC; dyeing in aniline blue for 1–3 min, rinsed by 1%HAC, dehydrated by ethanol gradient, clarified in xylene and fixed with neutral resin, and images were obtained under a microscope.

### Statistical analysis

The graphs were generated by Graph Pad Prism 7.0 software. Data were presented as mean ± standard deviation (SD). Statistical analyses were performed using SPSS 19.0 software. The t-test was used for comparison between two independent samples, and Comparisons among multiple groups were made by the one-way analysis of variance (ANOVA). P < 0.05 was considered statistically significant. All data were repeated in triplicate.

## Results

### Co-cultivation with senescent endothelial cells promotes the senescence of skin fibroblasts

**Fig. 1:**
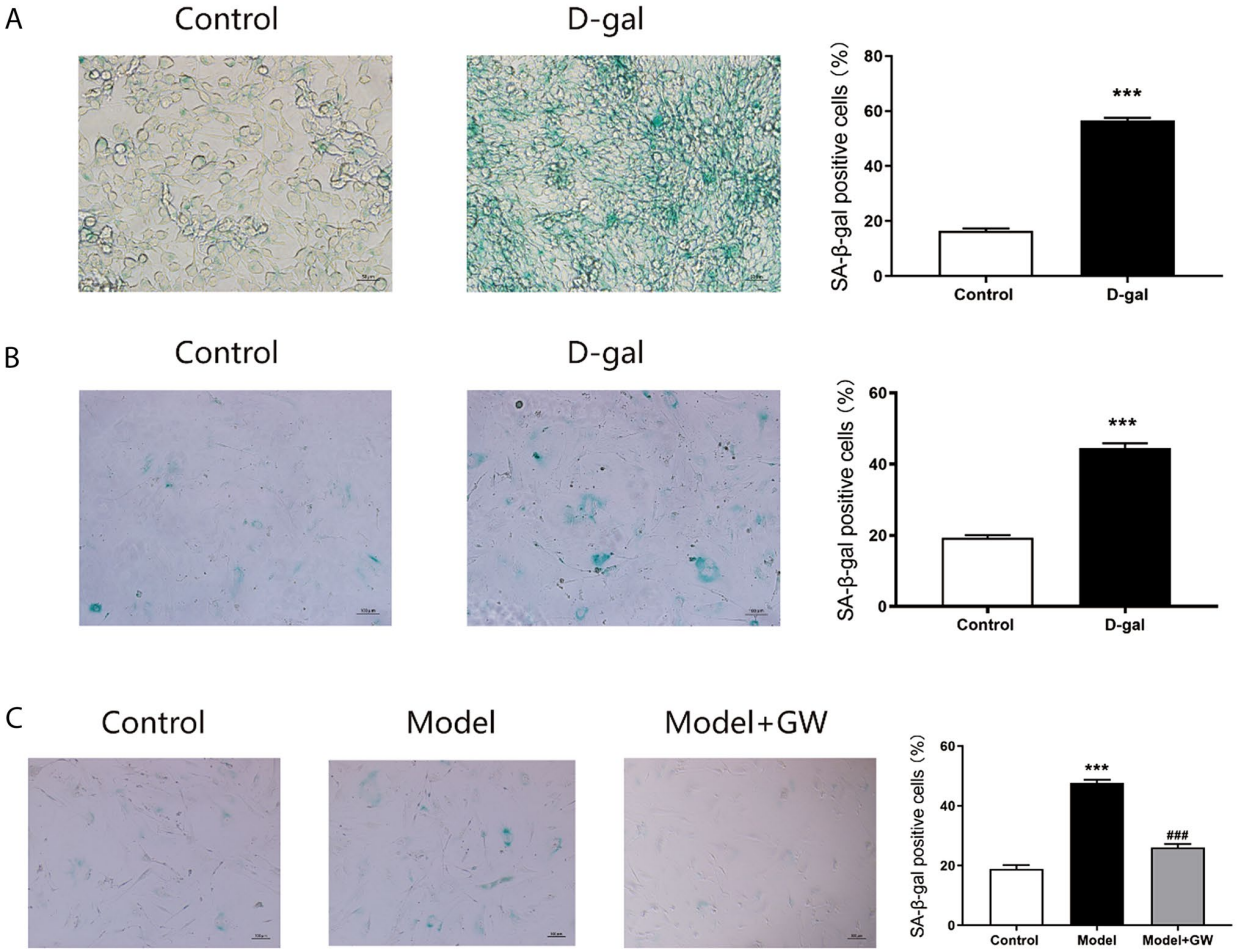
Staining of senescent cells. **A** SA-β-gal staining and positive cell number analysis of endothelial cells induced by D-gal for 48 h (200 ×). **B** SA-β-gal staining and positive cell number analysis of fibroblasts cells induced by D-gal for 48 h (100 ×). **C** SA-β-gal staining and positive cell number analysis of skin fibroblasts after co-culture with aging endothelial cells (Model) and GW4869 after co-culture (Model + GW) (100 ×)

To investigate whether vascular endothelial cells exosomes could regulate the fibroblasts aging process, we established a model of senescence in vascular endothelial cells via D-gal. The β‐galactosidase staining showed that 20 g/L D-galactose (D-gal) treatment significantly increased the positive number of senescent cells (Fig. 1A), indicating the success of the vascular endothelial cell senescence model. Then, β‐galactosidase staining of isolated cultured skin fibroblasts treated with 20 g/L D-gal showed a significant increase in the number of positive senescent cells (Fig. 1B). The co-culture system of endothelial cells and skin fibroblasts was established through Transwell chamber. Co-culture of skin fibroblasts with D-galactose treatment vascular endothelial cells (Model) revealed that senescent cells were increased by using β‐galactosidase staining in skin fibroblasts (Fig. 1C). GW4869 is an exosome secretion inhibitor. Vascular endothelial cells received treatment with D-gal and co-culture with skin fibroblasts, then added with GW4869 (Model + GW). We found that senescent cells were decreased (Fig. 1C). Thus, vascular endothelial cells-derived exosomes might play an essential role in regulating the skin fibroblasts aging process.

### miR-767 expression increases in exosomes of senescent endothelial cells

**Fig. 2:**
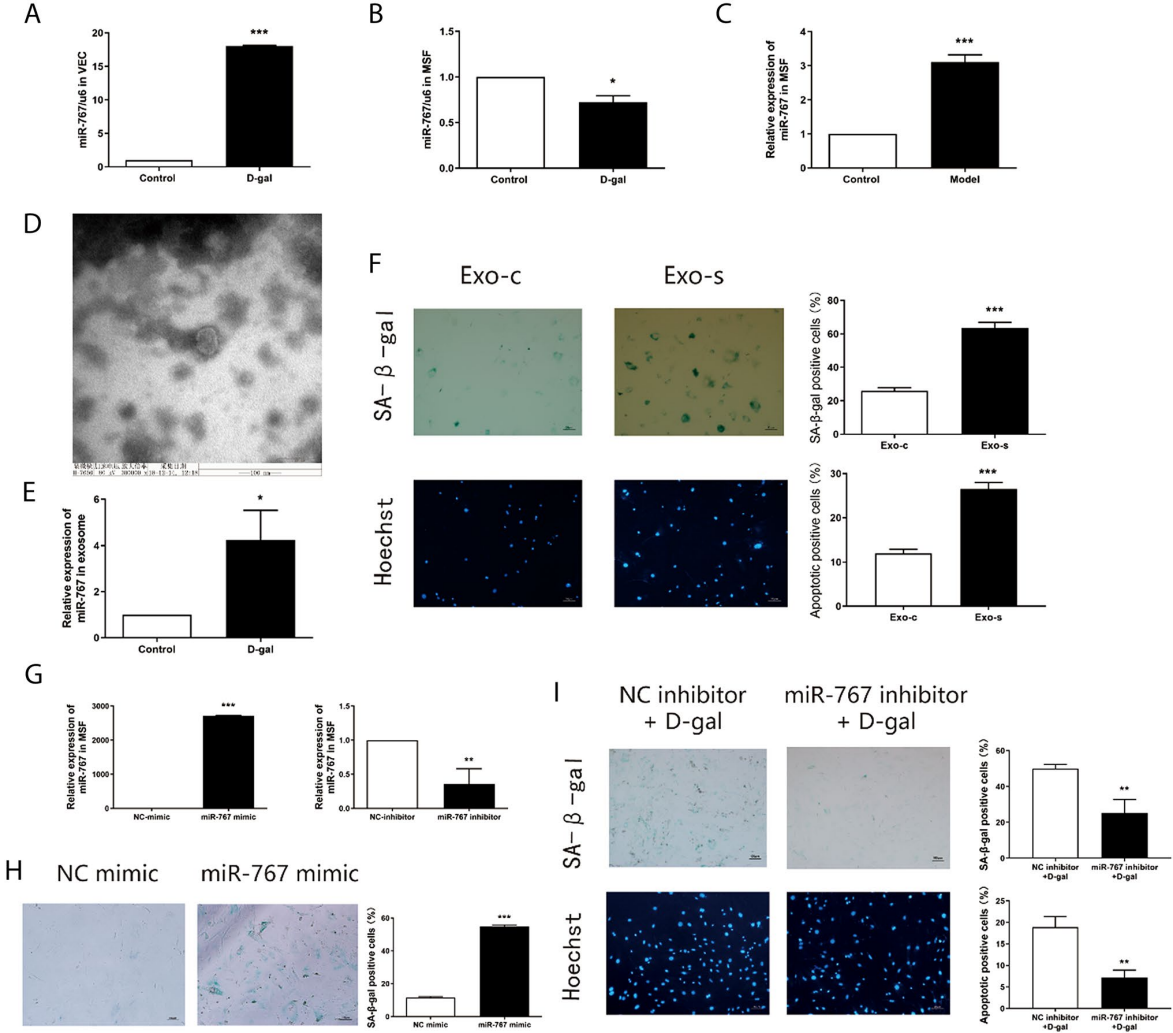
miR-767 expression in exosomes of senescent endothelial cells and regulates senescence in fibroblasts. **A** Expression of miR-767 in skin fibroblasts by qRT-PCR. **B** Expressions of miR-767 in vascular endothelial cells by qRT-PCR. **C** Expression of miR-767 in skin fibroblasts after co-cultured with senescent endothelial cells by qRT-PCR. **D** Exosomes derived from vascular endothelial cells were observed by ultraelectron microscopy. **E** Expression of miR-767 in exosomes derived from senescent vascular endothelial cells by qRT-PCR. **F** SA-β-gal staining and Hoechst staining of skin fibroblasts with senescent vascular endothelial cells derived exosomes (100 ×). **G** Expression of miR-767 after transfection with miR-767 mimic and miR-767 inhibitor in skin fibroblasts by qRT-PCR. **H** The ratio of positive cells in skin fibroblasts after transfection miR-767 mimic was detected by SA-β-gal staining (100 ×). **I** The ratio of positive cells in skin fibroblasts after transfection miR-767 inhibitor + D-gal was detected by SA-β-gal staining and Hoechst staining (100 ×)

As vascular endothelial cells-derived exosomes may carry miRNAs to exert their functions, then we screened senescence-related miRNAs from senile skin chip tissues previously. For example, miR-302-3p may promote skin fibroblast senescence by targeting inhibition the expression of AKT1 (Yang et al. 2020). We examined the expression of these miRNAs in separately cultured senescent skin fibroblasts and senescent vascular endothelial cells. Among them, miR-767 had aroused our attention. The expression of miR-767 was reduced in senescent skin fibroblasts (Fig. 2A). In contrast, the expression of miR-767 was significantly increased in senescent vascular endothelial cells (Fig. 2B). To further explore the relationship between aging vascular endothelial cells and aging skin fibroblasts, we determined miR-767 expression in the co-culture system of senescent endothelial cells and skin fibroblasts using qRT -PCR. The results showed that miR-767 in skin fibroblasts after co-cultured with senescent endothelial cells was significantly increased (Fig. 2C). Likewise, to explore the expression of miR-767 in senescent vascular endothelial cell exosomes. before that, exosomes derived from senescent vascular endothelial cells were isolated according to the instructions of the SBI Exosome Extraction Kit. vesicles in the shape of "saucer" were observed in the extract, with a diameter of about 60 nm (Fig. 2D) under scanning electron microscope. Additionally, the expression of miR-767 was significantly increased in senescent vascular endothelial cell exosomes via qRT-PCR (Fig. 2E).

### Exosomes of senescent endothelial cells accelerate fibroblasts senescence

In order to explore whether the exosomes of senescent vascular endothelial cells have the same functional effect on skin fibroblasts as they co-culture with senescent vascular endothelial cells. We separately added exosomes of non-senescent endothelial cells and exosomes of senescent endothelial cells to the skin fibroblasts. Turns out, senescent vascular endothelial cells derived exosomes significantly increased the number of senescence-positive cells and apoptosis-positive cells (Fig. 2F). These suggested that exosomes of senescent endothelial cells could accelerate fibroblasts senescence and apoptosis. As same as the functional effect on skin fibroblasts after co-culture with senescent endothelial cells.

### miR-767 regulates senescence in fibroblasts

To explore the functional role of miR-767 in fibroblasts, both gain and loss-of function experiments were performed in skin fibroblasts. First, skin fibroblasts were transfected with NC-mimic, miR-767 mimic, NC-inhibitor, miR-767 inhibitor to perform qRT -PCR to detect the transfection efficiency. miR-767 mimic increased the expression of miR-767 and miR-767 inhibitor suppressed the expression of miR-767 in skin fibroblasts (Fig. 2G). After miR-767 mimic was introduced, senescent cells were found to be significantly increased in skin fibroblasts (Fig. 2H). Following the inhibition of miR-767 expression in skin fibroblasts based on D-gal, senescent cells and apoptotic cells were decreased in skin fibroblasts (Fig. 2I). Consequently, skin fibroblasts lost the ability of aging vascular endothelial cell exosomes to promote senescence and apoptosis after inhibiting miR-767. These findings suggested that miR-767 could regulates senescence in fibroblasts.

### TAB1 is a target gene miR-767

**Fig. 3:**
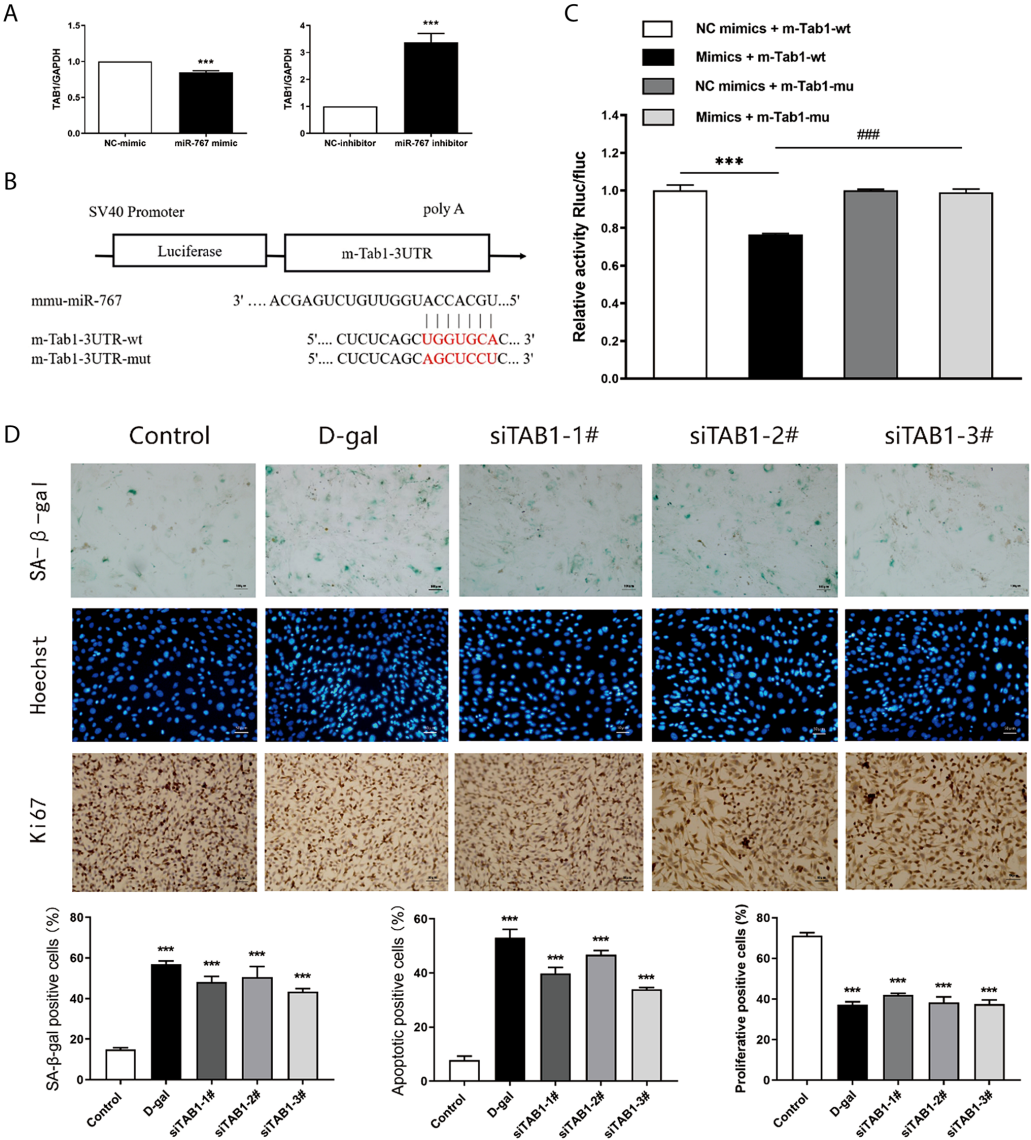
TAB1 affects senescence related biological functions of skin fibroblasts. **A** Expression of TAB1 after transfection with miR-767 mimic and miR-767 inhibitor in skin fibroblasts by qRT-PCR. **B** Schematic diagram of binding of mmu-miR-767 to m-TAB1-3UTR target sites. **C** The interaction between mmu-miR-767 and m-Tab1-3UTR was detected by Double Luciferase reporter gene. **D** Ratio of positive cells in siTAB1-treated skin fibroblasts stained with SA-β–gal staining (100 ×), Hoechst staining (100 ×) and Ki67 staining (200 ×)

The TargetScan database suggested that TAB1 may be the target gene. To validate the relationship between TAB1 and miR-767, TAB1 expression was determined by using qRT -PCR following transfection with miR-767 mimic and miR-767 inhibitor respectively. The results suggested that the mRNA expression of TAB1 in skin fibroblasts transfected with miR-767 mimic was decreased and the mRNA expression of TAB1 in skin fibroblasts transfected with miR-767 inhibitor was increased (Fig. 3A). Subsequently, a dual-luciferase reporter gene assay was performed to verify whether TAB1 was a target of miR-767. We found that luciferase activity in cells co-transfected with miR-767 mimic and TAB1-wt reporter plasmid was obviously declined. In contrast, co-transfection of TAB1-mut reporter plasmid and miR-767 mimic resulted in the complete retraction of reporter inhibition, indicating that miR-767 could bind to TAB1 mRNA (Fig. 3B, C). These confirmed that TAB1 was the target gene of miR-767. In addition, when TAB1 was silenced in skin fibroblasts, we found that compared with the control group senescence and apoptosis-positive cells increased, and proliferation-positive cells decreased (Fig. 3D). These results indicated that skin fibroblasts senescence and apoptosis was promoted, and proliferation was inhibited by TAB1 silencing.

### Expression of miR-767 and TAB1 in aging skin

**Fig. 4:**
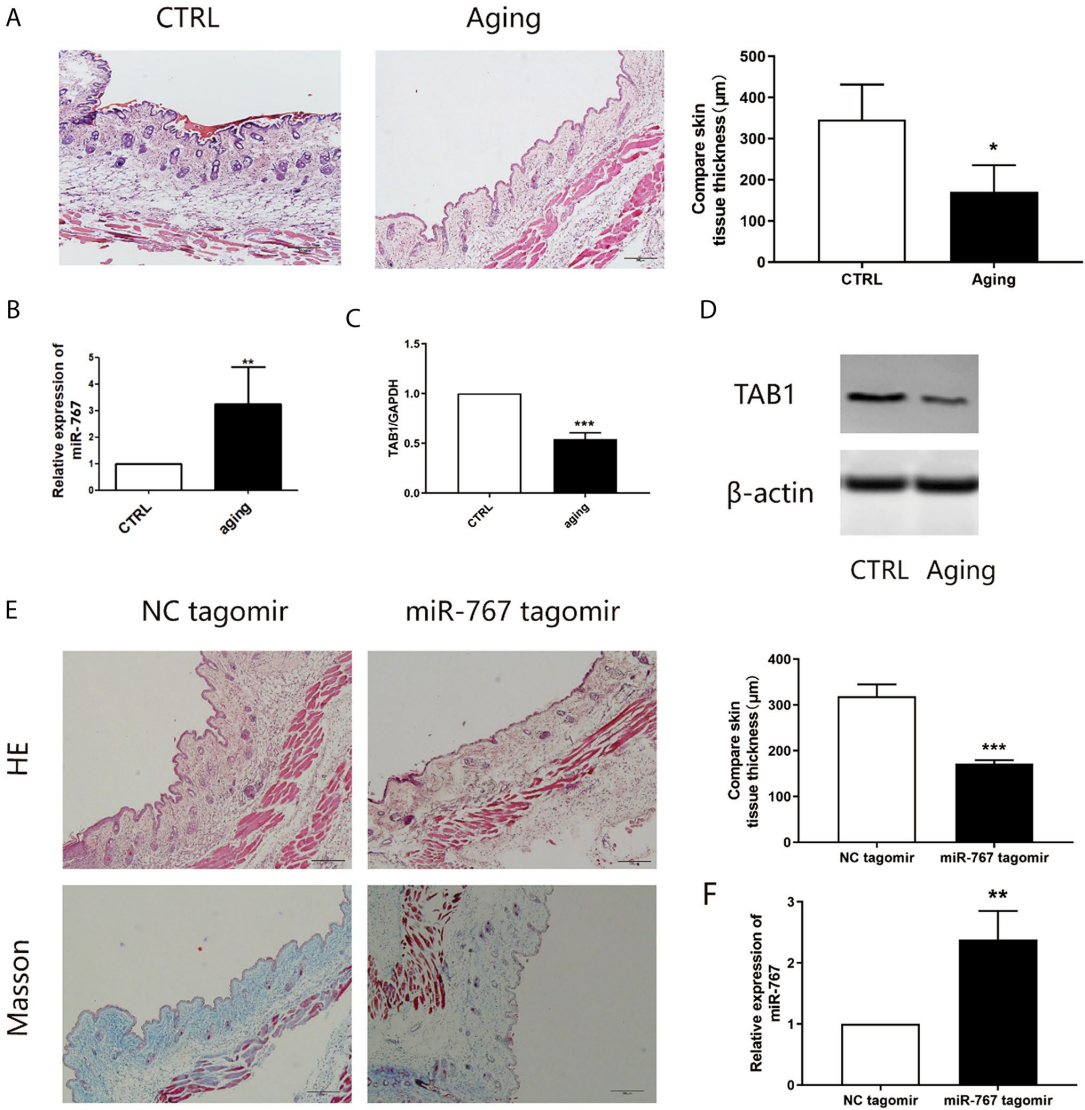
Analysis of miR-767 and TAB1 in aging skin. **A** HE staining for D-gal induced skin tissue(100 ×). **B** Expression of miR-767 in D-gal induced skin tissue by qRT-PCR. **C** Expression of TAB1 in D-gal induced skin tissue by qRT-PCR. **D** Expression of TAB1 in D-gal induced skin tissue by WB. **E** HE staining and Masson staining for miR-767 tagomir induced skin tissue(100 ×). **F** Expression of miR-767 in miR-767 tagomir induced skin tissue by qRT-PCR

Finally, in order to validate the effect of miR-767 on aging in mice. HE staining was performed on d-Gal induced mice to observe the epidermal thickness to detect the aging of mice. The results showed that the skin thickness of mice treated with D-Gal was significantly lower than that of mice treated with normal saline (Fig. 4A). Additionally, the expression of miR-767 and TAB1 detected by qRT-PCR showed that miR-767 was significantly increased and TAB1 expression was decreased in d-Gal treated mice (Fig. 4B, C), which was in consistent with the protein expression of TAB1 assessed using Western blot analysis (Fig. 4D). Next, we injected miR-767 mimics (miR-767 agomir group) to verify the role of miR-767 in vivo. HE staining and Masson staining were conducted, the epidermal thickness of mice treated with miR-767 mimics was significantly thinner, and the collagen layer was reduced (Fig. 4E). In the same way, we detected the expression of miR-767 in miR-767 mimics treated mice by qRT-PCR, which showed higher levels than mice treated with NC (Fig. 4F). These results suggest that overexpression of miR-767 may lead to aging manifestations.

## Discussion

Our results suggest that senescent vascular endothelial cells – derived exosome may deliver miR-767 to skin fibroblasts, and by inhibiting the expression of its target gene TAB1, thereby promoting skin fibroblasts aging, apoptosis, and inhibiting proliferation. In previous studies, we found that kelp polysaccharides may play a role in delaying skin aging by improving the function of vascular endothelial cells (Li et al. 2013a, b; Li et al. 2013a, b). In this study, through a co-culture system of vascular endothelial cells and skin fibroblasts, we found that vascular endothelial cells may accelerate the aging process of skin fibroblasts through the exosome pathway. Our discovery that senescent vascular endothelial cells regulate the aging of fibroblasts by secreting exosomes may help to discover new targets for delaying skin aging and achieve precision medical treatment.

We first found that, after establishing a co-culture system of vascular endothelial cells and skin fibroblasts through the Transwell chamber, senescent vascular endothelial cells may promote the aging and apoptosis of skin fibroblasts and inhibit cell proliferation. Interestingly, when exosome inhibitor GW4869 was added to senescent vascular endothelial cells, there was no significant change in the function of skin fibroblast senescence, apoptosis and cell proliferation. Studies have shown that bone marrow mesenchymal stem cells-derived exosomes increase the expression of miR-155 in nucleus pulposus cells (NPCs), and target bach1 to activate NPCs autophagy, inhibit apoptosis, and improve intervertebral disc degeneration (Shi et al. 2021). In addition, Yichao Wang et al. have found that miR-224-5p carried by human umbilical cord mesenchymal stem cells-derived exosomes (hUCMSCs-exo) can stimulate the proliferation and autophagy of breast cancer cells, while inhibit apoptosis (Wang et al. 2021a, b). We also speculate that senescent endothelial cells may regulate the biological functions of skin fibroblasts through exosomal pathways.

Exosomes are one of the smallest extracellular vesicles, which are involved in cell-to-cell communication and protein and RNA delivery (Hamdan et al. 2021), especially the molecular component miRNA, which has broad prospects as a new type of biomarker (Lin et al. 2015). In the skin microenvironment, intercellular communication and information transmission involving exosomes play an important role in maintaining cell function and tissue homeostasis (Yin et al. 2020). Studies have shown that exosomes are involved in the communication between various types of skin cells and are involved in the molecular pathogenesis of chronic inflammatory skin diseases (Wang et al. 2019). Besides, exosomes derived from human umbilical venous endothelial cells (HUVECs) accelerate skin wound healing and promote the proliferation and migration of keratinocytes and fibroblasts (Zhao et al. 2020). Exosomes derived from human induced pluripotent stem cells (iPSCs) can promote the proliferation and migration of aging human dermal fibroblasts (HDFs) in UVB-induced photoaging and natural aging models (Oh et al. 2018).

MicroRNAs (miRNAs) are a class of small non-coding RNAs that participate in the regulation of post-transcriptional gene expression and regulate various cell activities, such as cell growth, differentiation, development and apoptosis (Saliminejad et al. 2019). Recent studies have shown, miR-320a was overexpressed in cardiac fibroblasts derived exosomes, which can promote the proliferation of cardiac fibroblasts by promoting the PIK3CA/Akt/mTOR signaling pathway (Wang et al. 2021a, b). Elevated Bone marrow mesenchymal stem cell-derived exosomal miR-206 promotes the proliferation and differentiation of osteoblasts in osteoarthritis and inhibits apoptosis by reducing Elf3 (Huang et al. 2021). Interestingly, the mmu-miR-291a-3p derived from embryonic stem cells is an anti-aging factor that inhibits the cellular senescence of human dermal fibroblasts through the TGF-β receptor 2 pathway. Moreover, the results indicated that the ESC-derived exosomes mmu-miR-291a-3p have the potential for cell-free therapeutic interventions against aging and aging-related diseases (Bae et al. 2019). In our research, we discovered a large number of miRNAs related to aging through microarray analysis of aging skin tissues, such as miR-326-3p, miR-767, miR-155-5p, miR-34a-5p, miR-411-5p, miR-149-5p and so on. We explored the level of miR767 in senescent vascular endothelial cells—derived exosomes and their expression levels after co-culture with skin fibroblasts. miR-767 is highly expressed in co-cultured skin fibroblasts, and its overexpression promotes senescence, apoptosis and inhibits cell proliferation of skin fibroblasts. Similar research has shown that miR-767 promoted cell proliferation in human melanoma by suppressing CYLD expression (Zhang et al. 2018). Ultrasound targeted microbubble destruction (UTMD)-mediated miR-767 inhibition can further inhibit the proliferation, migration and invasion of non-small cell lung cancer (Li et al. 2020). However, the role of miR-767 in skin aging or body aging has not been reported.

microRNAs regulate cell evolution and metabolism through translational inhibition or mRNA degradation of post-transcriptional gene expression (Bartel. 2004; Yu et al. 2016). We found that TGF-beta activated kinase 1/MAP3K7 binding protein 1 (TAB1) is the target gene of miR-767 by TargetScan and other bioinformatics software databases. TAB1 is the upstream pathway protein of TAK1. TAB1 regulates cell senescence, apoptosis and proliferation through TAK1 signaling pathway. Furthermore, down-regulation of miR-873 can inhibits the proliferation of colorectal cancer cells by targeting TRAF5 and TAB1 (Gong et al. 2018), and miR-134 regulates the proliferation and apoptosis of ovarian cancer cells by regulating TAB1 gene expression (Shuang et al. 2017). Similar to elevated miR-767, inhibition of TAB1 can promote the senescence and apoptosis of skin fibroblasts, and inhibit cell proliferation. Therefore, our research shows that miR-767 can regulate the senescence, apoptosis and proliferation of skin fibroblasts by targeting TAB1.

There may be other mechanisms for vascular endothelial cells to regulate the function of skin fibroblasts. If we can establish a miR-767 overexpression model of vascular endothelial cells and then directly culture it with fibroblasts, this problem can be further explained. In addition, the mimics or inhibitors used in this study may be too strong to accurately represent the physiological effects of miR-767. Therefore, future studies should use exosomes isolated from the aged to determine whether the exosomes exhibit similar effects to the mimics and inhibitors used in this study.

In conclusion, we demonstrated that senescent vascular endothelial cells-derived exosomal miR-767 promotes skin fibroblast senescence, apoptosis and inhibits proliferation via targeting TAB1. This study provides new ideas and targets for delaying skin aging in the future, and miR-767 may become a target for delaying skin aging.

## Data Availability

The authors confirm that the data supporting the findings of this study are available within the article.
